# News in AL Amyloidosis ASH 2016: A rapidly evolving field of investigation

**DOI:** 10.1007/s12254-017-0332-6

**Published:** 2017-05-24

**Authors:** Hermine Agis

**Affiliations:** Department of Internal Medicine I, Division of Oncology, Medical University Vienna, Währinger Gürtel 10–20, 1090 Vienna, Austria

**Keywords:** Protein misfolding disease, Antifibrillary antibody, NEOD001, 11-1F4, Anti-SAP antibody

## Abstract

Amyloidosis is a rare but life-threatening protein misfolding disease. The early diagnosis and enrollment of patients into multicentre trials is of great importance, as is the need for intensive collaboration between multiple medical departments and experienced specialists. In the following review, the most interesting abstracts from the annual American Society of Hematology (ASH) meeting in 2016 are presented. The topics include the limitations of established biomarkers in risk assessment and response evaluation, the introduction of a new biomarker, the comparison of different treatment sequences and the efficacy of a multiple drug regimen in light-chain (AL) amyloidosis.

## Introduction

Systemic light-chain (AL) amyloidosis is a rare and life-threatening disease characterised by excessive light-chain depositions in the tissues and organs. The toxic light-chain paraprotein originates from clonal plasma cells or from a small B‑cell clone [1]. The most commonly involved organs are the heart, kidneys and the liver along with the gastrointestinal tract and the peripheral and autonomic nervous systems. The pattern of organ involvement determines the clinical presentation, and the symptoms are often very unspecific: fatigue, dyspnea, ankle and leg edema, weight loss, diarrhea, obstipation and orthostatic hypotension [2, 3]. However, there are some specific but rare signs and symptoms suggesting the presence of amyloidosis. These include periorbital ecchymosis, an enlarged tongue, bilateral carpal tunnel syndromes and nail dystrophy [2].

**Fig. 1:**
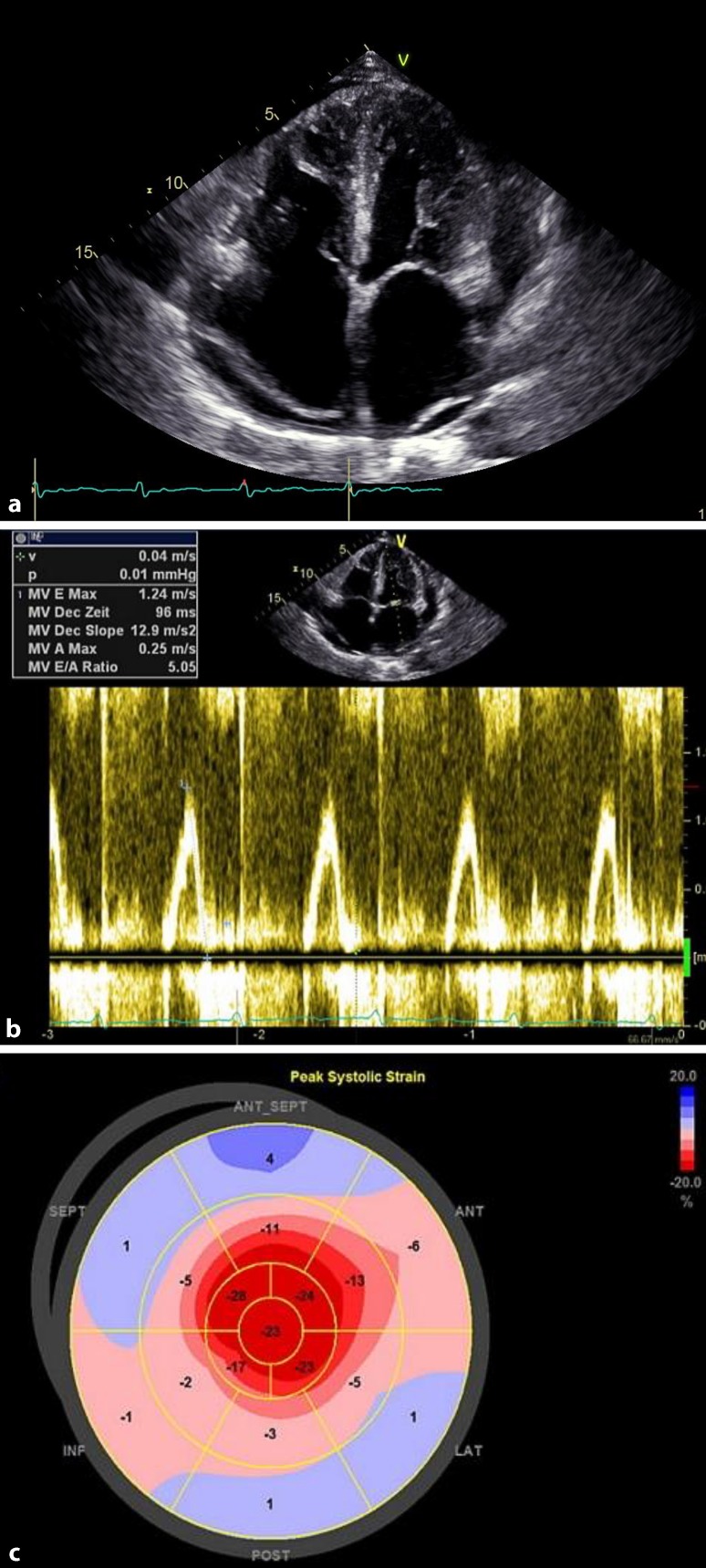
Transthoracic echocardiography. The typical features of cardiac amyloidosis in transthoracic echocardiography in a four-chamber view: a speckled appearance of the hypertrophied intraventricular septum, dilated atria and pericardial effusion (**a**). Pulsed-waved Doppler echocardiography illustrating a restrictive filling pattern with an E/A ratio of 5.05 (**b**). Two-dimensional speckle-tracking revealing the apical sparing of the longitudinal strain (**c**). Courtesy of Franz Ducka, MD (Medical University Vienna, Department of Internal Medicine II, Division of Cardiology)

**Fig. 2:**
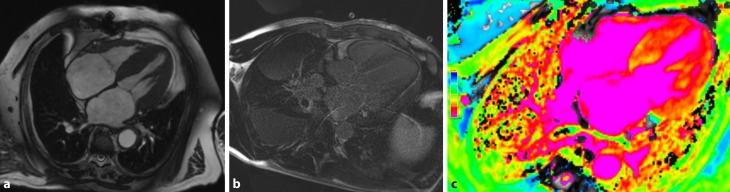
Cardiac MRI features in amyloidosis. The cine imaging shows hypertrophic left and right ventricles as well as severely dilated atria (**a**). Typical late enhancement pattern 15 min after gadolinium application (**b**). Native T1 mapping, which is used for extracellular volume quantification (native T1 time myocardium: 1180 ms; extracellular volume: 56.8%; **c**). Courtesy of Franz Ducka, MD (Medical University Vienna, Department of Internal Medicine II, Division of Cardiology)

**Fig. 3:**
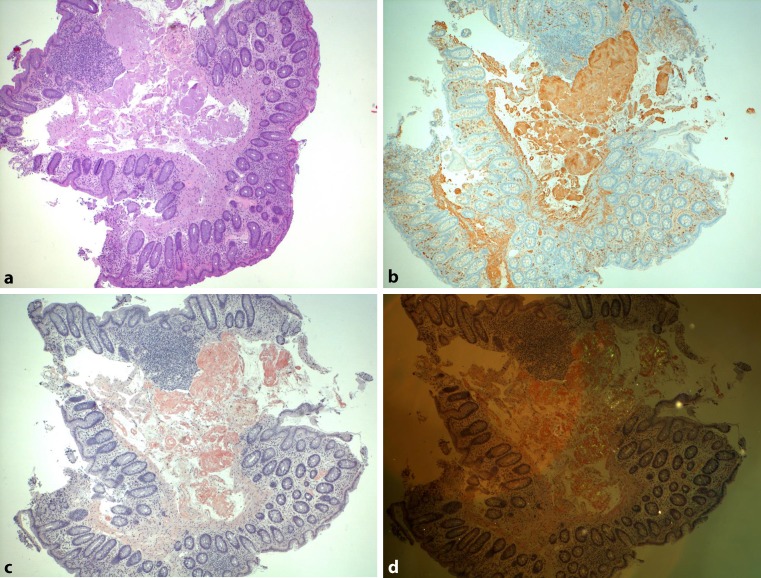
Colonic AL amyloidosis. **a** Histology and a hematoxylin and eosin (*H&E*) stain; original magnification (x10). Focal eosinophilic homogenisation of the submucosa. **b** Immunohistological amyloid typing with a kappa light-chain antibody revealing the specific positive staining of the deposits. **c** Congo red staining of the deposits with **d** characteristic apple-green birefringence under polarised light. Courtesy of Ingrid Simonitsch-Klupp, MD (Medical University Vienna, Institute of Pathology)

Cardiac involvement is the most limiting survival factor [4], emphasising the need for early diagnosis. This can be accomplished with easily accessible biomarkers, mainly N‑terminal pro-brain natriuretic peptide (NT-proBNP; elevation >332 ng/l; in the absence of renal failure or atrial fibrillation) and albuminuria (>0.5 g in 24 h; in the absence of long-lasting diabetes or hypertension). These markers have been indicated in published studies as useful for risk assessment prior to therapy and for response evaluation [5]. Furthermore, NT-proBNP and albuminuria are important biomarkers in amyloidosis screening, especially when combined with noninvasive imaging techniques like echocardiography and cardiac MRI (Figs. 1 and 2). The final diagnosis has to be verified by a mandatory biopsy, with the exception of ATTR amyloidosis (transthyretin amyloidosis, mutant and wild types). In a first step, Congo red staining has to be positive for amyloid deposits. As a next step, immunohistochemistry (IHC) has to be carried out to characterise the origin of the amyloid fibrils ([6]; Fig. 3). Fibril typing is a crucial process within the diagnostic workflow because different types of amyloidosis need different treatment strategies. If IHC is not successful in defining the underlying protein, laser capture microdissection and mass spectroscopy should be performed [7, 8].

### Problems in risk assessment and response evaluation with established biomarkers

For risk assessment before the treatment initiation and subsequent response evaluation, testing for the cardiac biomarkers NT-proBNP and troponin T is recommended, as is the measurement of systolic blood pressure [9, 10]. For the renal risk classification, proteinuria >5 g/24 h and an estimated glomerular filtration rate (eGFR) < 50 ml/min [11, 12] are of prognostic significance. The response evaluation is of great importance because organ improvement is achieved only along with haematological response. NT-proBNP is used as a main determinant of cardiac response assessment, and the US Food and Drug Administration (FDA) considers this biomarker the primary end point in clinical trials. However, NT-proBNP levels are markedly influenced by renal function, fluid overload and drug interactions that are not further specified. The optimal time points (6 or 12 months) of NT-proBNP evaluation during the course of the disease remain unclear. To address this problem, an observational prospective trial was initiated [13]. NT-proBNP values that were analysed at 6 months did not result in a significant impact on overall survival (OS). However, evaluating NT-proBNP at 6 months resulted in a misclassification of nearly half of all potential cardiac responders. Only NT-proBNP levels captured at 12 months demonstrated prognostic significance regarding OS. This study highlights the critical importance of the timing of NT-proBNP measurements in the response assessment for clinical trials to avoid false negative results.

### Evaluation of a new biomarker

Growth differentiation factor-15 (GDF-15) is a protein belonging to the transforming growth factor beta superfamily. It acts as a pleiotropic cytokine involved in the regulation of inflammatory and apoptotic pathways in injured tissues. Kastritis et al. [14] investigated GDF-15 levels in the peripheral blood in two independent cohorts at two different centres (Pavia and Athens). Independently of cardiac biomarkers and renal involvement, elevated GDF-15 levels >7575 pg/ml were associated with early death, shorter survival times and faster progression to dialysis.

### First-line treatment

So far, no approved (by the FDA or the European Medicines Agency, EMA) treatment is available for AL amyloidosis. Chemotherapy, proteasome inhibitors, immunomodulatory drugs (IMiD) and autologous stem cell transplantation (ASCT) are used only in an off-label manner. Some centres prefer upfront ASCT as an induction therapy, and other institutions use front-line therapy (bortezomib-based, ± IMiDs and corticosteroids) [15] in order to achieve organ improvement before ASCT. To address the question of which strategy provides the best outcome, bortezomib-based first-line treatment preceding ASCT was investigated in various trials, and the results were reported at the ASH meeting. Kastritis et al. presented the first prospective randomised phase III trial of melphalan and dexamethasone (MDex) versus bortezomib, melphalan and dexamethasone (BMDex) for newly diagnosed patients [16]. Alahwal et al. also presented data regarding the high effectiveness of bortezomib-containing regimens in these patient cohorts [17]. Both trials identified bortezomib as an effective drug with profound haematological responses and organ improvement. However, the study designs and end points were different, and a direct comparison of these two trials is therefore not applicable. Interestingly, both working groups mentioned a bortezomib-based cardiac toxicity. Therefore, Alahwal et al. emphasised the importance of a nonbortezomib-based approach for patients with advanced cardiac dysfunction, i. e. melphalan/dexamethasone [18] to avoid additional toxicity, since these patients were found to have an inferior survival rate [17].

Sidana et al. introduced a trial comparing a bortezomib-containing regimen with a nonbortezomib-containing induction regimen in transplant ineligible patients with AL amyloidosis [19]. Again, the treatment with bortezomib-based regimens resulted in better responses, including higher rates of early very good partial remission (VGPR) at 3 and 6 months. The patients achieved faster cardiac improvement and had a lower relapse rate, but no difference in OS was seen.

Two further trials with bortezomib-based induction therapy followed by ASCT were presented. Jain et al. performed a retrospective data analysis, while Minnema et al. did the data collection in a prospective designed trial. Both anlyses demonstrated an improved outcome with a better OS [20, 21]. In comparing these trials, it should be mentioned that the data collection was retrospectively in the working group of Jain et al. and in a prospective manner in Minnema’s group. However, both trials showed the beneficial effects of early ASCT after bortezomib-containing induction therapy.

The group led by Tandon investigated standard-dose versus risk-adapted melphalan conditioning on the outcome in patients undergoing front-line ASCT [22]. Melphalan dose reduction is used in patients with older age, renal dysfunction and multiorgan involvement. The reduced dosage (140–160 mg/m^2^) compared to the standard dose of melphalan (200 mg/m^2^) resulted in a suboptimal outcome [21], suggesting that high-dose therapy should be limited to patients eligible for full-dose conditioning.

### Biochemical patterns of relapse in AL amyloidosis

In relapsed AL amyloidosis, we face the same problems as in newly diagnosed patients: there is no standard of care and validated progression criteria and treatment recommendations are still lacking. However, since several new therapeutic options are available for plasma cell dyscrasias, new data on the effectiveness of these drugs are also being awaited in connection with AL amyloidosis. Three abstracts at the ASH meeting in 2016 were of special interest.

Tandon et al. investigated the predictors of early relapse during initial therapy [23]. It was demonstrated that patients with early relapse are of older age with a higher prevalence of cardiac and multiorgan involvement and a higher risk stage (Mayo III and IV). Therefore, early ASCT should be an aim during the initial therapy. Milani et al. described the relapse patterns after different upfront strategies [24] in 259 patients. A low free light chain (FLC) burden at diagnosis and a bortezomib-containing induction regimen were associated with more durable responses. Cardiac deterioration was preceded by increasing dFLC (the difference between involved and uninvolved free light chains) in >90% of patients. An increase of >20% in dFLC should be a clear trigger to start with the relapse therapy. A “wait and see” strategy until the NT-proBNP levels increase will result in a worsened course of disease. Hwa et al. evaluated the relapse patterns after ASCT and their influence on the disease outcome [25]. The following scenarios were evaluated: (1) hematologic relapse only, (2) organ progression only and (3) combined hematologic and organ relapse. As expected, the OS rates 4 years after ASCT were 87.8% for hematologic relapse, 63.9% for organ relapse and 56.7% (*p* = 0.0016) for combined organ and hematologic progression.

### New therapeutic options in relapsed patients

Lagos et al. presented data from a phase II trial with bendamustine combined with dexamethasone. The regimen was well tolerated with significant success in hematologic response rates in previously treated AL amyloidosis [26]. Cohen et al. investigated the effects of carfilzomib [27] in the relapse setting. Carfilzomib was feasible and effective, including in bortezomib pretreated patients, with a maximal tolerated dose (MTD) of 20 mg/m^2^ at the first infusion followed by a dosage of 36 mg/m^2^ as a 30 min infusion. However, cardiac, pulmonary and renal toxicities were documented. The close monitoring of blood pressure with the avoidance of hypertensive episodes during and after the drug infusion is recommended. NT-proBNP levels may rise without a direct correlation with progressive cardiac dysfunction. The same effect is observed during IMiD therapy. The mechanism of this phenomenon is not fully understood. Increasing NT-proBNP levels in these special settings has to interpreted with great caution. Kaufmann et al. presented data on the use of daratumumab in heavily pretreated patients with cardiac involvement [28]. In this retrospective single centre study, antibody treatment with daratumumab was very well tolerated, resulting in rapid and significant hematologic responses. After a median time of 4 months of therapy, cardiac dysfunction improved markedly. Other mAbs with different modes of action were also investigated, as listed below.

- The first additional study involved antifibrillary mAbs directed against epitopes expressed only by misfolded amyloid fibrils. Gertz et al. [29] investigated NEOD001 in the PRONTO trial (a phase I/II dose-escalation trial). NEOD001 is a humanised mAb binding to a cryptic epitope unique to misfolded light-chain fibrils. This epitope is exposed during misfolding and aggregation and is not targetable in regular conformation. This mAb not only disrupts amyloid fibrils, but also stimulates macrophages to phagocyte amyloid deposits. Patients eligible for this study had completed at least one round of previous therapy, with at least a partial hematologic response or better, and suffer from persistent organ dysfunction. NEOD001 was administered intravenously every 28 days to the 27 patients enrolled. No drug-related serious adverse events (AEs) were documented; the patients reported only fatigue, upper respiratory tract infection, nausea and diarrhea at a maximum of grade I or II (which was not clearly related to the study drug). No discontinuations were reported. Eight of 14 patients with cardiac involvement met the criteria for cardiac response (measured as the percentage of decrease in the pro-BNP levels), and 9 of 15 patients with renal involvement met the criteria for renal response (measured as the percentage of decrease in proteinuria in g/24 h). Because of these promising results, an expansion cohort was initiated, and the follow-up results were presented at the ASH meeting in 2016 [30]. Forty-two additional patients were enrolled, including 16 patients with renal involvement, 15 patients with cardiac involvement and 11 patients with peripheral nerve amyloidosis. Again, no dose-limiting toxicities or discontinuations were reported. Of the cardiac-affected patients, 53% met the criteria for organ response along with 63% of renal-affected patients (expressed as the percentage of decrease in NT-proBNP or proteinuria in g/24 h); none of the patients experienced disease progression. The median times to the initial response were 2 months (cardiac) and 4 months (renal). After 9 months of treatment, 82% of patients with peripheral neuropathy achieved a response based on the Neuropathy Impairment Score in the Lower Limbs (NIS-LL score).
  Edwards et al. [31] presented the results of a chimeric amyloid fibril reactive mAb 11-1F4 mediating amyloid destabilisation and the initiation of cell-mediated phagocytosis. The first data collected in a phase Ia trial were presented at the ASH meeting in 2015. At the ASH meeting in 2016, the follow-up results from a phase Ia/b trial were introduced [31]. The mAb 11-1F4 was administered without severe adverse events (only fatigue, upper respiratory tract infection, nausea and diarrhea), and no dose-limiting toxicities up to an MTD of 500 mg/m^2^ were documented. The clinical efficacy resulted in an early and sustained organ response.
- The second additional study involved an anti-SAP mAb directed against the ubiquitously present SAP (Serum Amyloid P) component. The anti-SAP mAb works only in conjunction with the small molecule drug CPHPC, which was constructed for the binding and elimination of circulating SAP molecules from the blood stream. In a second step, the anti-SAP mAb was administered intravenously. This antibody binds to the residual SAP molecules located in the tissue amyloid deposits, triggering rapid amyloid clearance through complement activation and macrophage stimulation. The first results with this new drug combination were presented at the ASH meeting in 2014. In 2015, Richards et al. published this open-labelled, single-dose escalation, phase I trial titled “Therapeutic Clearance of Amyloid by Antibodies to Serum Amyloid P Component” in the *New England Journal of Medicine* [32]. This phase I trial involved 15 patients with amyloidosis (two with AA amyloidosis, eight with AL amyloidosis, four with AFib amyloidosis and one with AApoA1 amyloidosis). The affected organs were the kidney, liver, spleen and lymph nodes. Patients with cardiac involvement were strictly excluded. No serious side effects were reported with the anti-SAP antibody and CPHPC. A response assessment was conducted after 6 weeks with a SAP scan using the ^123^I-SAP scintigram and a FIBRO scan. After 6 weeks, decreased liver stiffness was detected by FIBRO scan and by ^123^I-SAP scan. In one patient improvement in the liver function tests were measured. The same reduction was seen in the spleen and in the affected lymph nodes also documented by the SAP scan [32].

#### Take-home messages

AL amyloidosis is a life-threatening systemic disease requiring early diagnosis with accessible biomarker measurements. For the final diagnosis, a multidisciplinary approach by specialised and experienced teams is strongly recommended. Enrolling patients in prospective, randomised international trials should still be the goal because no therapeutic standard option has been approved so far. However, an increasing number of trials with promising monoclonal antibodies and small molecules are being conducted in specialised amyloidosis centres.

## References

[1] Kyle RA, Gertz MA (1995). Primary systemic amyloidosis: clinical and laboratory features in 474 cases. Semin Hematol.

[2] Mahmood S, Palladini G, Sanchorwala V (2014). Update on treatment of light chain amyloidosis. Haematologica.

[3] Merlini G, Belotti V (2003). Molecular mechanism s of amyloidosis. N Engl J Med.

[4] Merlini G, Palladini G (2013). Light chain amyloidosis: the heart of the problem. Haematologica.

[5] Merlini G, Wechalekar AD, Palladini G (2013). Systemic light chain amyloidosis an update for treating physicians. Blood.

[6] Picken MM (2010). Amyloidosis where are we now and where are we heading?. Arch Pathol Lab Med.

[7] Vrana JA, Gamez JD, Madden BJ (2009). Classification of amyloidosis by laser microdissection and mass spectrometry-based proteomic analysis in clinical biopsy specimen. Blood.

[8] Brambilla F, Lavatalli F, Di Silvestre D (2012). Reliable typing of systemic amyloidosis through proteomic analysis of subcutaneous adipose tissue. Blood.

[9] Dispenzieri A, Gertz MA, Kyle RA (2004). Serum cardiac troponins and N‑terminal pro-brain natriuretic peptide: a staging system for primary systemic amyloidosis. J Clin Oncol.

[10] Kumar S, Dispenzieri A, Lacy MQ (2012). Revised prognostic staging system for light chain amyloidosis incorporating cardiac biomarkers and serum free light chain measurements. J Clin Oncol.

[11] Merlini G, Lousada I, Ando Y (2016). Rationale, application and clinical qualification for NT-proBNP as a surrogate end point in pivotal clinical trials in patients with AL amyloidosis. Leukemia.

[12] Palladini G, Hegenbart U, Milani P (2014). A staging system for renal outcome and early markers of renal response to chemotherapy in AL amyloidosis. Blood.

[13] Wechalekar A, Foard D, Whelan C (2016). Challenges of Using NT-ProBNP for response assessment in aystemic AL amyloidosis – Analysis of a prospective study. Blood.

[14] Kastritis E, Merlini G, Papassotiriou I (2016). Growth Differentiation Factor-15 (GDF-15) Is a new biomarker with independent prognostic significance for survival and renal outcomes in different cohorts of patients with light chain (AL) Amyloidosis. Blood.

[15] Dispenzieri A, Buadl F, Kumar SK (2015). Treatment of immunoglobulin light chain amyloidosis. Mayo Clin Proc.

[16] Kastritis E, Leleu X, Arnulf B (2016). A randomized phase III trial of Melphalan and Dexamethasone (MDex) versus Bortezomib, Melphalan and Dexamethasone (BMDex) for untreated patients with AL Amyloidosis. Blood.

[17] Alahwal H, Song KW, Duggan P (2016). Bortezomib-containing regimens for the treatment of newly diagnosed AL Amyloidosis. Blood.

[18] Palladini G, Milani P, Foli A (2014). Oral melphalan and dexamethasone grants extended survival with minimal toxicity in AL amyloidosis: long-term results of a risk-adapted approach. Haematologica.

[19] Sidana S, Tandon N, Dispenzieri A (2016). Bortezomib versus non-Bortezomib based treatment for transplant ineligible patients with light chain Amyloidosis. Blood.

[20] Jain T, Kung ST, Shah V (2016). Treatment with Bortezomib-based therapy followed by Autologous stem cell transplantation improves outcomes in light chain Amyloidosis. Blood.

[21] Minnema M, Nasserinejad K, Hazenberg B (2016). Results of 25 patients from a multicenter, multinational, prospective phase II study of Bortezomib based induction treatment followed by Autologous stem cell transplantation in patients with newly diagnosed Al Amyloidosis. Blood.

[22] Tandon N, Sidana S, Dispenzieri A (2016). Standard dose versus risk adapted Melphalan conditioning on outcomes in systemic AL Amyloidosis patients undergoing frontline autologous stem cell transplant based on revised mayo stage. Blood.

[23] Tandon N, Sidana S, Gertz MA (2016). Predictors of Early Relapse (ER) following initial therapy for systemic immunoglobulin light chain Amyloidosis. Blood.

[24] Milani P, Basset M, Russo F (2016). Patterns of relapse after upfront therapy in AL Amyloidosis. Blood.

[25] Hwa YL, Warsame R, Gertz MA (2016). Practice patterns of re-initiation of therapy at time of relapse or progression post-autologous stem cell transplant among patients with AL Amyloidosis. Blood.

[26] Lagos GG, Lentzsch S, Comenzo RL (2016). Final results of a phase 2 study of Bendamustine in combination with Dexamethasone in patients with previously treated systemic light-chain Amyloidosis. Blood.

[27] Cohen AD, Landau H, Scott EC (2016). Safety and efficacy of Carfilzomib in previously treated systemic light-chain Amyloidosis. Blood.

[28] Kaufmann G, Witteles R, Wheeler M (2016). Hematologic responses and cardiac organ improvement in patients with heavily pretreated cardiac immunoglobulin light chain (AL) Amyloidosis receiving Daratumumab. Blood.

[29] Gertz MA, Landau H, Commenzo RL (2016). First-in-human phase I/II study of NEOD001 in patients with light chain Amyloidosis and persistent organ dysfunction. J Clin Oncol.

[30] Gertz MA, Comenzo RL, Landau H (2016). NEOD001 demonstrates organ biomarker responses in patients with light chain Amyloidosis and persistent organ dysfunction: results from the expansion cohort of a phase 1/2 study. Blood.

[31] Edwards CV, Gould J, Langer AL (2016). Analysis of the phase 1a/b study of chimeric fibril-reactive monoclonal antibody 11-1F4 in patients with AL Amyloidosis. Blood.

[32] Richards DB, Cookson LM, Berges AC (2015). Therapeutic clearance of Amyloid by antibodies to serum Amyloid P component. N Engl J Med.

